# LncRNA linc00312 suppresses radiotherapy resistance by targeting DNA-PKcs and impairing DNA damage repair in nasopharyngeal carcinoma

**DOI:** 10.1038/s41419-020-03302-2

**Published:** 2021-01-04

**Authors:** Zhen Guo, You-Hong Wang, Heng Xu, Chun-Su Yuan, Hong-Hao Zhou, Wei-Hua Huang, Hui Wang, Wei Zhang

**Affiliations:** 1 Department of Clinical Pharmacology, Xiangya Hospital, Central South University, Changsha, 410008 China; 2 Institute of Clinical Pharmacology, Hunan Key Laboratory of Pharmacogenetics, Central South University, Changsha, 410078 China; 3 National Clinical Research Center for Geriatrics, Xiangya Hospital, Central South University, Changsha, 410008 China; 4 Academician Workstation, Changsha Medical University, Changsha, 410219 China; 5 Department of Laboratory Medicine, National Key Laboratory of Biotherapy/Collaborative Innovation Center of Biotherapy and Cancer Center, West China Hospital, Sichuan University, Chengdu, 610000 China; 6 Tang Center for Herbal Medicine Research, University of Chicago, Chicago, IL 60637 USA; 7 Department of Anesthesia and Critical Care, University of Chicago, Chicago, IL 60637 USA; 8 Key Laboratory of Translational Radiation Oncology, Hunan Province; Department of Radiation Oncology, Hunan Cancer Hospital and The Affiliated Cancer Hospital of Xiangya School of Medicine, Central South University, Changsha, 410013 China

**Keywords:** Head and neck cancer, Non-homologous-end joining, Long non-coding RNAs, Prognostic markers

## Abstract

Radioresistance is the main obstacle in the clinical management of nasopharyngeal carcinoma (NPC). linc00312 is deregulated in a number of human cancers, including NPC. However, the detailed functions and underlying mechanisms of linc00312 in regulating radiosensitivity of NPC remains unknown. In this study, cox regression analysis was used to assess the association between linc00312 and NPC patients’ survival after radiotherapy. Our results reveal that linc00312 is significantly down-regulated in NPC tissues and patients with higher expression of linc00312 are significantly associated with longer overall survival and better short-term radiotherapy efficacy. Overexpression of linc00312 could increase the sensitivity of NPC cells to ionizing radiation, as indicated by clonogenic survival assay, comet assay, and flow cytometry. Mechanistically, RNA pull down and RNA immunoprecipitation were performed to investigate the binding proteins of linc00312. linc00312 directly binds to DNA-PKcs, hinders the recruitment of DNA-PKcs to Ku80, and inhibits phosphorylation of AKT–DNA–PKcs axis, therefore inhibiting the DNA damage signal sensation and transduction in the NHEJ repair pathway. In addition, linc00312 impairs DNA repair and cell cycle control by suppressing MRN–ATM–CHK2 signal and ATR–CHK1 signal. In summary, we identified DNA-PKcs as the binding protein of linc00312 and revealed a novel mechanism of linc00312 in the DNA damage response, providing evidence for a potential therapeutic strategy in NPC.

## Introduction

Nasopharyngeal carcinoma (NPC) is a malignant tumor originating from the epithelial cells of the nasopharynx with highly metastatic and invasive features^1^. Currently, radiotherapy is the primary treatment modality for NPC. Approximately 70% of newly diagnosed patients with NPC are at advanced stages and are more likely to suffer treatment failure from radiotherapy due to radioresistance—with recurrence (10~20%) and metastasis (20~30%)—which is the leading cause of NPC mortality^2^. Radioresistance is also the main obstacle in the clinical management of NPC. Therefore, it is vital to identify the molecular mechanism of NPC radioresistance and develop a radiosensitization strategy for anticancer therapies.

Long non-coding RNA (lncRNA) is widely involved in regulating numerous physiological and pathological processes in cells at three levels—transcriptional, post-transcriptional, and epigenetic—and is closely related to the occurrence, development, and prognosis of cancers^3^. In recent years, it has been reported that lncRNA is also involved in regulating radiosensitivity in many types of tumors by handling DNA damage response, cell stemness, epithelial to mesenchymal transition (EMT), cell cycle arrest, and apoptosis^4–7^. Still, very little is known about the radiosensitive role of lncRNAs specific to NPC.

linc00312 is an intergenic lncRNA located on 3p25.3 and first found in NPC. Deletion at 3p25.3 is commonly found in NPC, indicating this region may involve NPC-associated tumor suppressors^8^. Interestingly, Huang C et al. demonstrated linc00312 had a dual effect on NPC cells as it inhibited cell proliferation but promoted cell invasion^9^. The authors also indicated that positive expression of linc00312 was associated with a good prognosis in patients with NPC, with no lymph node metastasis^10^. Only one study reported linc00312 enhanced the sensitivity of ovarian cancer cells to cisplatin by promoting cell apoptosis^11^. Until now, the role of linc00312 in cancer development and progression was poorly understood. The functions and mechanisms of linc00312 in drug response and radiation response are only beginning to emerge and are yet to be elucidated.

In this study, we first assess the prognostic role of linc00312 in patients with NPC receiving radiotherapy. Then, by using in vitro and in vivo experiments, we determine the effect of linc00312 on radiosensitivity in NPC cells. Finally, we identify the direct binding partner of linc00312 and unveil the underlying mechanism in radiation response regulation, thereby providing new insights for irradiation therapeutic strategies.

## Results

### linc00312 is down-regulated in NPC and associates with poor radiotherapy efficacy

**Fig. 1:**
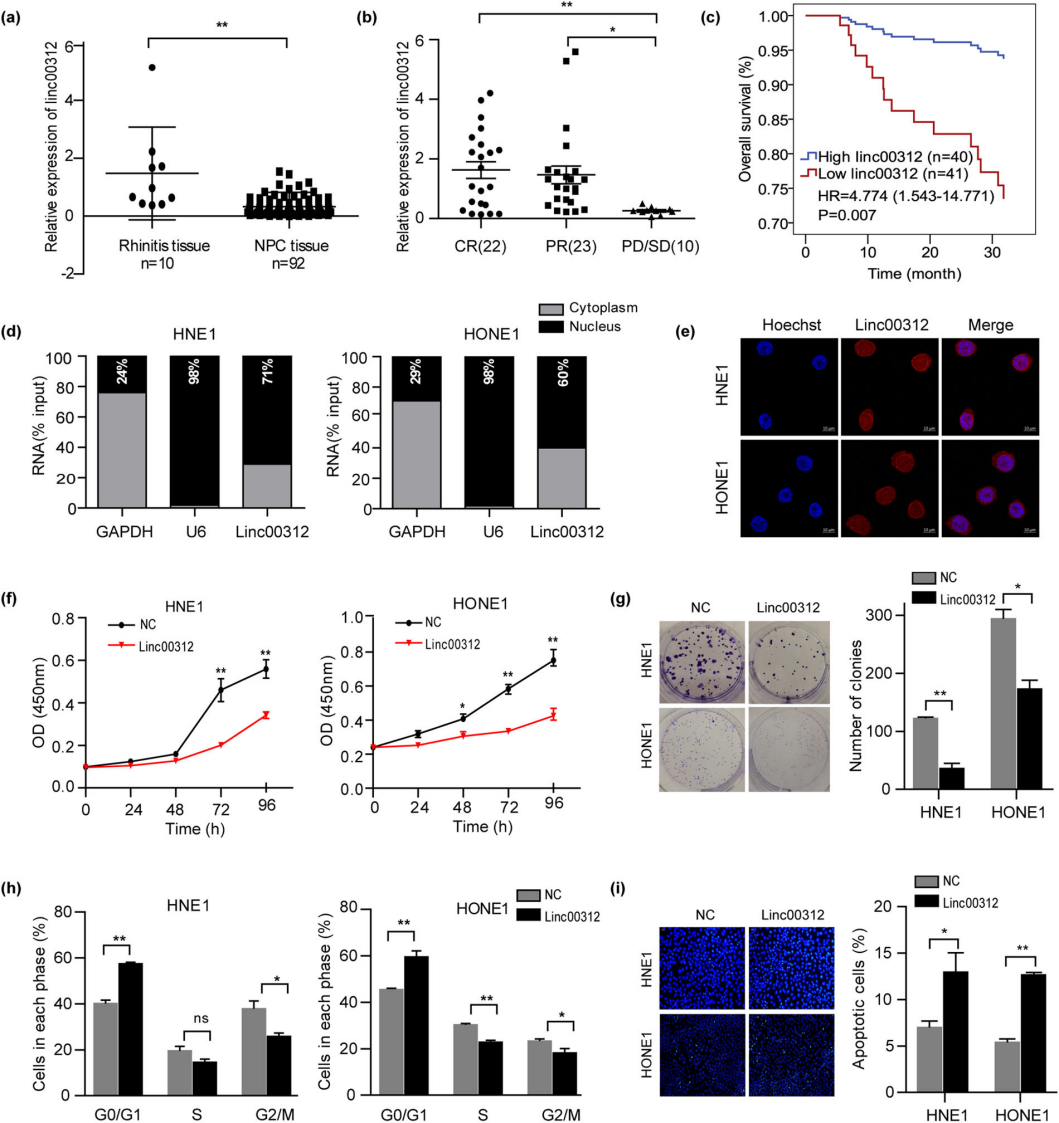
linc00312 is down-regulated in NPC and functions as a tumor suppressor. **a** The expression of linc00312 was detected by real-time RT-PCR in 92 NPC tissue samples and 10 normal nasopharyngeal tissue samples with chronic inflammation. **b** Real-time RT-PCR was used to determine the expression of linc00312 in patients with NPC, with radiosensitive response (PR/CR**)** and radioresistance response (PD/SD). **c** Cox regression analysis of the association between linc00312 expression level and NPC patients’ 3-year overall survival. **d** The subcellular localization of linc00312 in HNE1 and HONE1 cells was detected by cytoplasm/nuclear separation assay followed by real-time RT-PCR. GAPDH was utilized as the control of cytoplasm, and U6 was utilized as the control of nuclear. **e** Representative images of FISH showing the subcellular localization of linc00312 in HNE1 and HONE1 cells. **f** Cell viability of NPC cells transfected with linc00312 overexpression vector or control vector were measured by CCK-8 assay in series of time points. **g** The colony-formation ability of NPC cells transfected with linc00312 overexpression vector or control vector were detected by colony formation assay. **h** Flow cytometry was used to detect the cell cycle of NPC cells transfected with linc00312 overexpression vector or control vector. **i** The apoptosis of NPC cells transfected with linc00312 overexpression vector or control vector were measured by Hoechst staining. ^*^P < 0.05, ^**^P < 0.01.

We observed that linc00312 had a lower expression level in NPC tissues compared to chronic inflammation nasopharyngeal tissues (Fig. 1a). In addition, the expression level of linc00312 in patients with radiosensitive response (CR/PR) was significantly higher than in patients with radioresistance response (PD/SD) (Fig. 1b). The 3-year overall survival rate was significantly lower in patients who have a lower expression level of linc00312 (HR = 4.774, *P* = 0.007) (Fig. 1c). However, we failed to find an association between expression level of linc00312 and clinical stages of NPC (Supplementary Fig 1a).

### Ectopic expression of linc00312 inhibits NPC cell viability and promotes cell apoptosis

We first examined the subcellular localization of linc00312 in NPC cells and found that linc00312 was localized in both the nucleus and cytoplasm of NPC cells, with the nucleus having a higher proportion about 60% to 71% (Fig. 1d, e). The expression level of linc00312 was obviously lower in NPC cell lines (CNE1, CNE2, HONE1, and HNE1) by comparison to the normal nasopharyngeal epithelial cell line NP69, indicating a tumor suppressor role of linc00312 in NPC (Supplementary Fig 1b). Cells with ectopic overexpression of linc00312 showed a decreased cell viability in a time-dependent pattern (Fig. 1f and Supplementary Fig 1c). In addition, overexpression of linc00312 impaired the colony-formation ability of NPC cells (Fig. 1g). Flow cytometry analysis indicated that linc00312 overexpression might result in cell cycle arresting in the G0/G1 phase (Fig. 1h). Moreover, the fraction of apoptotic cells was significantly increased among the linc00312 overexpressed cells (Fig. 1i).

### linc00312 overexpression improves radiosensitivity of NPC cells in vitro

**Fig. 2:**
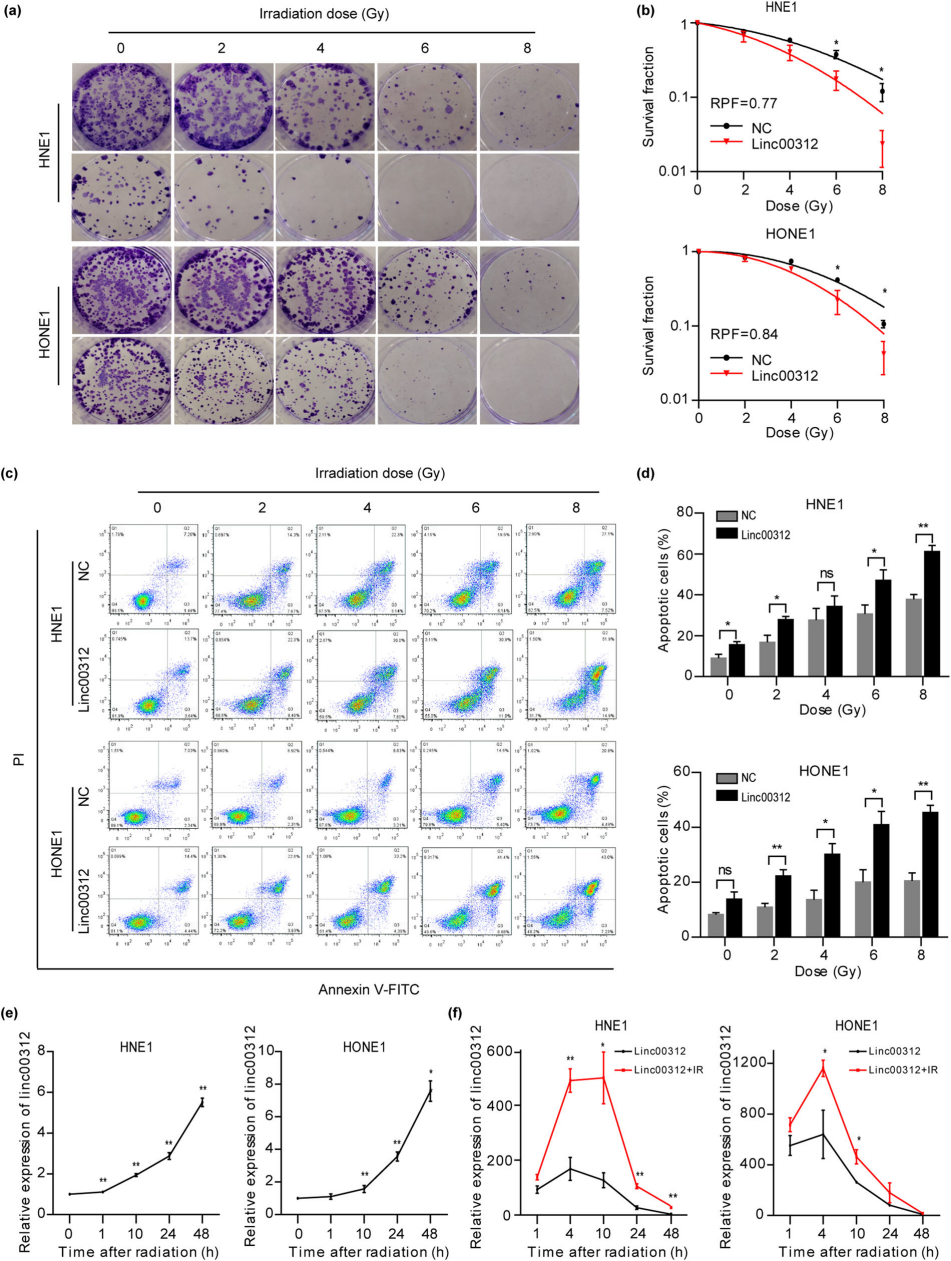
linc00312 overexpression improves radiosensitivity of NPC cells in vitro. **a** The clonogenic survival assay was used to determine the radiosensitivity of NPC cells. **b** The irradiation dose–survival curve was fitted using the single-hit multitarget model. **c** Cells were exposed to a series of irradiation dose, and flow cytometry was used to detect cell apoptosis. **d** The histogram of cell apoptotic rate in different treatment groups. **e** Real-time RT-PCR was used to detect the expression of linc00312 in NPC cells after 8 Gy irradiation at different points of time. **f** The irradiation-induced linc00312 overexpression effect was more remarkable if the cells were transfected with linc00312 overexpression vector before irradiation. ^*^P < 0.05, ^**^P < 0.01.

The clonogenic survival assay was used to assess cell sensitivity to radiation treatment. The survival colonies of the linc00312 overexpression group were significantly decreased compared to the control group in an irradiation dose-dependent manner (Fig. 2a). The radiation dose–survival curve showed that the cells transfected with linc00312 overexpression vector significantly increased the sensitivity of NPC cells to radiation treatment compared to the control vector (radiation protection factor; RPF_HNE1_ = 0.77; RPF_HONE1_ = 0.84) (Fig. 2b). Consistently, the apoptotic cell rate detected by flow cytometry was higher in the linc00312 overexpression group compared to the control group, and this trend was irradiation dose-dependent (Fig. 2c, d).

An interesting phenomenon observed by chance was that irradiation might induce linc00312 overexpression. After a dose of 8 Gy irradiation, the expression level of linc00312 in NPC cells gradually increased over time (Fig. 2e). Furthermore, by transfecting the cells with linc00312 vector or control vector before irradiation, the expression level of linc00312 was remarkably increased in the irradiation treatment group. To be precise, 4 h after irradiation, the expression level of linc00312 in HNE1 cells was 490 times higher in the linc00312 + IR group compared to 169 times higher in the linc00312 overexpression group (Fig. 2f).

### linc00312 overexpression enhances the sensitivity of xenograft tumor to radiation in vivo

**Fig. 3:**
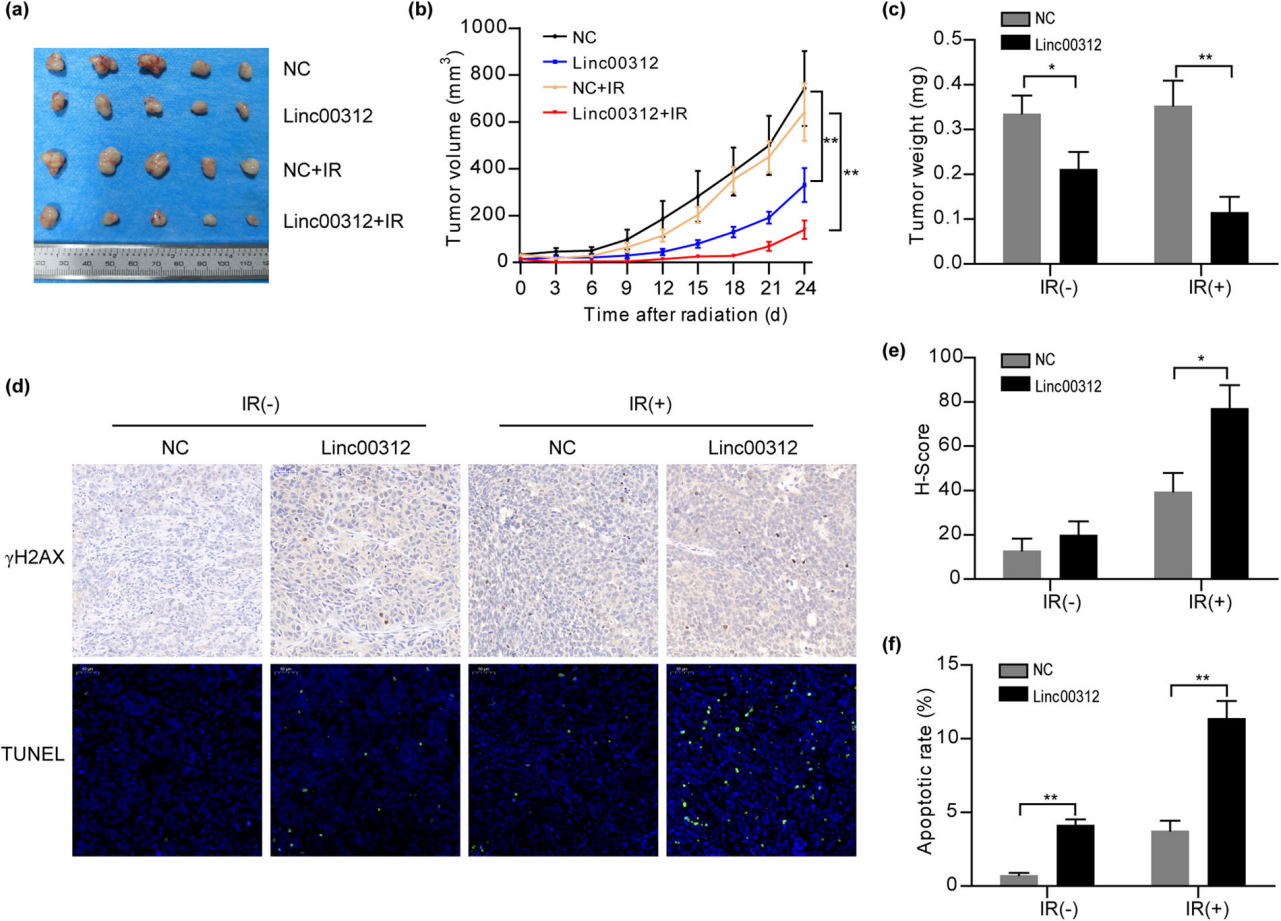
linc00312 overexpression enhances the sensitivity of xenograft tumor to radiation in vivo. **a** The isolated xenograft tumors from four groups with different treatments (linc00312 overexpression, control, linc00312 overexpression + 6 Gy irradiation, control + 6 Gy irradiation). **b** The tumor growth and volume in different treatment groups were measured every 3 days. **c** The weight of tumor in different treatment groups were weighted when the mice were sacrificed. **d** Representative images of γH2AX detected by immunohistochemical analysis and apoptotic cells detected by TUNEL assay in tumor sections from different treatment groups. **e** H-score was used to indicate the γH2AX-positive cells in tumor sections from different treatment groups. **f** The apoptotic rate detected by TUNEL assay was calculated by optical density. ^*^P < 0.05, ^**^P < 0.01.

To further investigate the in vivo radiosensitization effect of linc00312 in NPC, we established subcutaneous tumors in nude mice using HNE1 cells that stably express linc00312 or control lentiviral vector. The growth of tumor xenografts was significantly inhibited and the weight of tumors was significantly decreased in the linc00312 overexpression group compared to the control group, indicating the tumor suppressor role of linc00312. After 6 Gy of ionizing radiation, tumor growth and weight were further inhibited in the linc00312 + IR group compared to the control + IR group (Fig. 3a–c). Since γH2AX was considered a DNA damage marker, immunohistochemical analysis was carried out to detect γH2AX in tumor tissue sections. Compared with the control + IR group, more positive cells of γH2AX were present in the linc00312 + IR group (Fig. 3d, e). Moreover, TUNEL assay showed that more apoptotic cells were noted in the linc00312 + IR group compared to the control + IR group (Fig. 3d, f). Taken together, these results indicated that overexpression of linc00312 could sensitize NPC cells to ionizing radiation in vivo.

### linc00312 binds to the catalytic subunit of DNA-dependent protein kinase (DNA-PKcs)

**Fig. 4:**
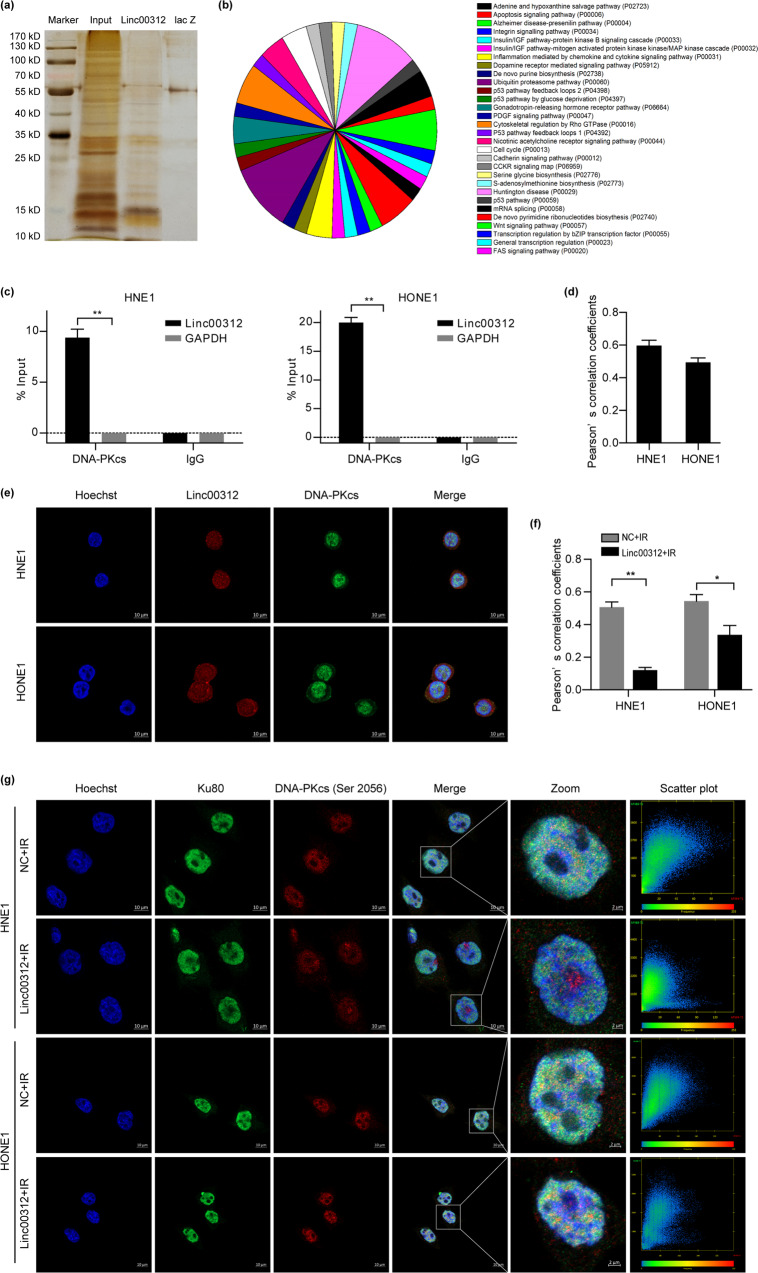
linc00312 directly binds to DNA-PKcs and impairs the formation of DNA–PK complex in response to DSBs. **a** The silver-stained PAGE gel that showed the separated proteins pulled down by linc00312. **b** Pathway enrichment analysis of the pulled down proteins. **c** The IP products from RIP assay were quantified by real-time RT-PCR. % Input reflects the enrichment efficiency of target genes in IP group compared with Input group. **d** Pearson’s correlation coefficients of images of internalized linc00312 and DNA-PKcs in NPC cells. **e** Representative confocal images of linc00312 (red) and DNA-PKcs (green) in NPC cells. **f** Pearson’s correlation coefficients of images of internalized DNA-PKcs (Ser2056) and Ku80 in NPC cells. **g** Representative confocal images of DNA-PKcs (Ser2056) (red) and Ku80 (green) at the indicated time points in linc00312 overexpressed cells or control cells that were treated with 8 Gy irradiation. The last column shows the scatterplot of red and green pixel intensities of NPC cells in different treatment groups. ^*^P < 0.05, ^**^P < 0.01.

One way that lncRNA executes its function is through interaction with specific binding proteins. However, until now, little was known about linc00312’s binding protein. To address this, we first conducted RNA pull-down assay using the biotin-labeled linc00312 transcript followed by mass spectrometry. We identified a number of proteins that specifically bind to linc00312 but not the control probe, among which DNA-PKcs showed the highest score (Fig. 4a). The mass spectrogram of DNA-PKcs was showed in Supplementary Fig 1d. The pathway enrichment analysis revealed that most of the pull-down proteins were enriched in ubiquitin proteasome pathway, apoptosis signaling pathway, and Wnt signaling pathway (Fig. 4b). To verify the interaction between linc00312 and DNA-PKcs, we performed RIP-PCR assay using DNA-PKcs-specific antibody and linc00312-specific primers. As expected, results from agarose gel electrophoresis and RT-PCR demonstrated that linc00312 was enriched in the anti-DNA-PKcs group compared to the control IgG group (Fig. 4c and Supplementary Fig 1e). To further confirm this interaction, we examined the co-localization of DNA-PKcs and linc00312 in NPC cells by immunofluorescence and fluorescence in situ hybridization (FISH). We found that 60% and 49% DNA-PKcs were co-localized with linc00312 in HNE1 and HONE1 cells, respectively (Fig. 4d, e). These findings revealed that DNA-PKcs was the specific binding protein of linc00312 in NPC cells.

### linc00312 inhibits recruitment of DNA-PKcs to Ku80 in response to DSBs

In response to DNA double strand breaks (DSBs), the Ku70-Ku80 heterodimer recognizes the DSBs and binds to the DNA ends first, forming a clamp-like complex that recruits DNA-PKcs to the Ku-DNA ends^12^. DNA-PKcs undergoes autophosphorylation at Ser2056 and causes conformational changes, which is essential for efficient rejoining of DSBs^13^. We have identified that DNA-PKcs specifically bound to linc00312, according to the RNA pull-down and RIP results. To determine whether or not linc00312 would affect DNA–PK complex formation in the occurrence of ionizing radiation, we performed immunofluorescence staining of DNA-PKcs (Ser2056) and Ku80. After exposing to 8 Gy irradiation, the co-localization coefficient between DNA-PKcs (Ser2056) and Ku80 was decreased in the linc00312 overexpressed cells, indicating that linc00312 inhibited recruitment of DNA-PKcs to Ku80 (Fig. 4f, g).

### linc00312 affects radiosensitivity by regulating the DNA damage repair pathway

**Fig. 5:**
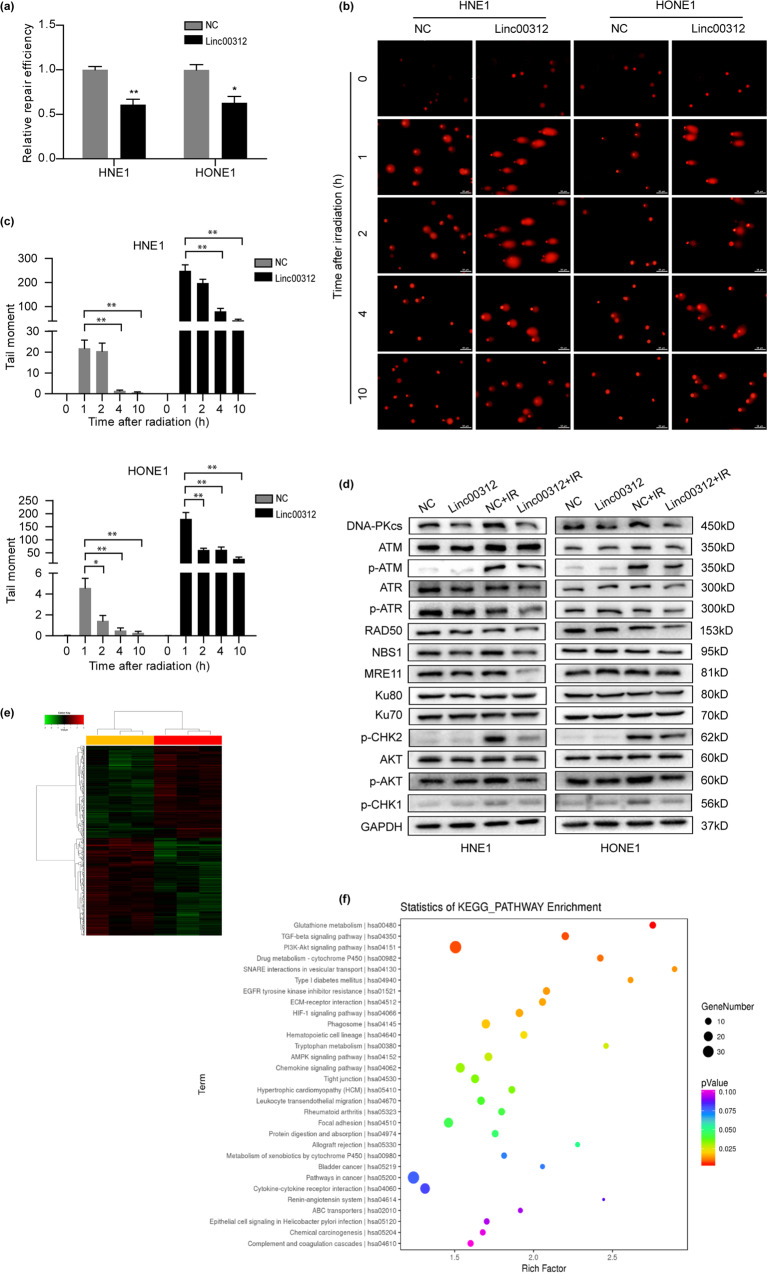
linc00312 affects radiosensitivity through regulating DNA damage response pathways. **a** The DNA repair efficiency was detected by NHEJ reporter assay. **b** Representative images of comet assay in NPC cells transfected with linc00312 overexpression vector or control vector at the indicated time points. **c** Ionizing radiation-induced DNA damage was quantified by the tail moment. **d** Western blotting of key proteins involved in DNA damage repair pathway that were affected by linc00312. **e** The clustering diagram of the six NPC cell samples: orange represents the three cell samples that received 8 Gy irradiation after transfection of linc00312 overexpression vector; red represents the three cell samples that received 8 Gy irradiation after transfection of control vector. **f** The differential genes (*P* < 0.05) identified by microarray between linc00312 + IR and control + IR groups were enriched by KEGG pathway analysis and displayed as bubble map. ^*^P < 0.05, ^**^P < 0.01.

Given that linc00312 binds to DNA-PKcs, a critical protein involved in the non-homologous end joining (NHEJ) pathway, we hypothesized that linc00312 might play a role in DNA DSB repair. Therefore, we first detected the DNA repair efficiency by NHEJ reporter assay. We found the cells with overexpression of linc00312 showed a decreased repair efficiency compared with the control cells (Fig. 5a). Moreover, we examined the effect of linc00312 on the repair of ionizing radiation-induced DNA damage by using comet assays following a series of time points. While the tail moment gradually returned to the baseline in the control cells 24 h after ionizing radiation, it remained high in the linc00312 overexpressed cells, indicating that DNA repair was delayed in cells with linc00312 overexpression (Fig. 5b, c). These results suggest that DSB-repair ability is impaired by overexpression of linc00312.

Also, we observed that overexpression of linc00312 significantly decreased the level of DNA-PKcs, and this trend was more remarkable after ionizing radiation. However, overexpression of linc00312 had no effect on Ku70/Ku80 expression. Ataxia telangiectasia mutated (ATM), and ATM and Rad-3 related (ATR) play an important role in DNA damage signal transduction and cell cycle regulation. CHK1 and CHK2, which can be activated by ATM/ATR kinase, play an important role in DNA damage checkpoint control and tumor inhibition^14^. In our study, we revealed the expression levels of p-ATM, p-ATR, p-CHK1, and p-CHK2 in the linc00312 overexpression + IR group were decreased compared to the control + IR group. The MRN complex (MRE11, RAD50, and NBS1) is activated after DNA damage and plays a crucial role in sensing, processing, and repairing DNA DSBs in the homologous recombination (HR) pathway. We found that expression levels of RAD50, MRE11, and NBS1 in the linc00312 + IR group were significantly lower than that in the control + IR group (Fig. 5d).

We also detected the expression of apoptosis-related proteins, finding increased expression of DNA damage marker γH2AX and pro-apoptotic molecule BAX and decreased expression of anti-apoptotic molecule BCL2 in the linc00312 + IR group compared to the control + IR group. Moreover, expression of c-PARP was increased in the linc00312 overexpression group after radiation treatment (Supplementary Fig 1f).

Next, we carried out RNA microarray in NPC cells to explore the genes and pathways affected by linc00312 after exposing to 8 Gy irradiation (Fig. 5e). We identified 364 down-regulated RNAs and 388 up-regulated RNAs in linc00312 overexpressed cells compared to control cells after irradiation (Fold change >1.3). We found most of the differently expressed mRNAs (*P* < 0.05) were enriched in the PI3K–AKT signaling pathway (Fig. 5f). It is known that ionizing radiation can activate the PI3K pathway, and activation of AKT leads to activation of DNA-PKcs. With this in mind, we contemplated AKT’s involvement in linc00312-mediated DNA-PKcs regulation. Consequently, we examined the expression of AKT and found that p-AKT was significantly decreased in the linc00312 + IR group compared to the control + IR group.

## Discussion

Radiation therapy is an important component of cancer treatment and especially vital in the treatment of NPC. The life quality of patients with NPC, with advanced stages is seriously threatened by radioresistance response. In the present study, we identified linc00312 as a radiosensitizer in NPC through regulating DNA damage response by directly binding to DNA-PKcs.

We confirmed the tumor suppressor role of linc00312 in NPC by inhibiting cell growth and promoting cell apoptosis. Also, in clinical samples, patients with NPC, with higher expression of linc00312 showed a better radiotherapy short-term efficacy and a better 3-year overall survival. In the next step, we determined the effect of linc00312 on NPC cell radiosensitivity. The clonogenic survival assay is considered the gold standard in determining cell sensitivity to radiation treatment. Ionizing radiation primarily targets on cell DNA and results in double strand breaks, which are considered a major cause of radiation-induced apoptosis. Therefore, the production of DSBs and cell apoptosis could be regarded as radiosensitivity to a certain degree. By conducting clonogenic survival assay, comet assay, flow cytometry, and xenograft model, we were able to demonstrate that linc00312 increased the sensitivity of NPC cells to radiation therapy.

Moreover, we found an interesting feedback loop between irradiation and linc00312. On the one hand, irradiation might induce linc00312 overexpression. On the other hand, overexpression of linc00312 might increase cell sensitivity to irradiation. This phenomenon was also reported in other lncRNAs. For example, the expression level of lncRNA PVT1 was significantly increased after irradiation treatment, in turn, overexpression of PVT1 contributed to radioresistance in NSCLC cells^15^.

We questioned how linc00312 exerts its function in regulating the radiation response? The intracellular localization of linc00312 in NPC cells distributed mainly in the nucleus, which is similar to its distribution in lung cancer cells^16^. LncRNA acts in a variety of ways by interacting with chromatin, protein, and mRNA. However, the exact molecular mechanisms of linc00312 are largely unknown and only two studies to date have reported the binding protein of linc00312. For example, linc00312 could directly bind to the transcription factor YBX1 and induce lung adenocarcinoma metastasis and vasculogenic mimicry^17^. Another study reported linc00312 could inhibit NSCLC cell proliferation and promote apoptosis by directly binding to the transcription factor HOXA5^16^. Here, by conducting RNA pull-down and RIP assay, we identified a new binding protein of linc00312, namely DNA-PKcs.

DNA-PKcs is the key kinase and plays a central role in the NHEJ pathway^12^. In mammalian cells, 80~90% of DSBs are repaired by NHEJ. Suppression of DNA-PKcs severely affected the repair capacity of the NHEJ pathway, leading to the sensitization of a variety of tumor cells to radiation^18^. It has been reported that lower expression of DNA-PKcs was associated with a better response after radiotherapy for NPC^19^. Positive DNA-PKcs nuclear staining was closely associated with biochemical recurrence in prostate cancer cells after radiotherapy^20^. DNA-PKcs alone is inactivated and relies on Ku to guide it to the DSB ends and trigger its kinase activity. The formation of DNA–PK complex provides a scaffold for other DNA repair components and ultimately leads to DSB ligation^12^. In this study, we demonstrated that linc00312 not only inhibited the expression of DNA-PKcs, but also hindered its recruitment to Ku-DNA ends, thereby impairing the NHEJ pathway. This mechanism is very similar to lncRNA LINP1 as LINP1 enhances the repair efficiency of the NHEJ pathway by serving as a scaffold to link Ku80 and DNA-PKcs, thereby decreasing sensitivity to radiotherapy in breast cancer patients^21^.

DNA damage responses are orchestrated by multiple signal transduction processes, key among which are the ATM–CHK2 and ATR–CHK1 pathways. Activation of these pathways is crucial for the proper coordination of checkpoint and DNA repair processes^22^. MRN complex plays a central role in ATM activation by recruiting ATM to DNA lesions and stimulating ATM kinase activity in response to DSB^23^. Autophosphorylation on Ser1981 is essential for ATM activation as it causes the dissociation of dimer ATM into active monomers^24^. The activated ATM phosphorylates a variety of downstream targets, including γH2AX, p53, NBS1, and CHK2, thereby controlling both cell cycle arrest and cell death pathways^25^. The MRN complex and ATM promote DNA-end resection at DSBs, allowing replication protein A (RPA) binding and activation of the ATR and CHK1 kinases that orchestrate checkpoint responses and allow replication to resume^26^. Lee et al. have shown a direct and nearly linear relationship between the levels of the MRN complex and the level of ATM phosphorylation^27^. Our results indicated that overexpression of linc00312 down-regulated MRN expression, leading to decreased ATM–CHK2 and ATR–CHK1 signal transduction. Nonetheless, the exact mechanisms through which linc00312 downregulate MRN in response to DSB remains to be elucidated. Increasing evidence have shown that deregulation of these proteins may have a predictive value for cancer prognosis. For example, overexpression of MRN correlated with poor disease-free survival and overall survival in patients with rectal cancer, receiving neoadjuvant radiotherapy^28^. Knockdown of RAD50 sensitized NSCLC cells to radiation and RAD50 expression was associated with distant relapse-free survival after radiotherapy for NSCLC patients^29^. Molecular inhibitors of ATM/ATR, for example, AZD1390 and VX-970, have been observed to result in both chemosensitivity and radiosensitivity in a wide range of cancer cells^30,31^.

It is well known that four classic signal transduction pathways participate in irradiation-induced DNA damage signal transduction: PI3K/AKT, MAPK/ERK, NF-κB, and TGF-β. These pathways may modulate tumor radioresistance by manipulating cell cycle checkpoints, DNA damage repair, and apoptosis^14^. Previous studies have identified aberrant constitutive activation of PI3K/AKT signaling in NPC^32^. Excessive activation of the PI3K/AKT signal contributes to radiation resistance of tumor cells and inhibition of this way is a promising modality to overcome radioresistance^33^. The pathway enrichment analysis indicated the PI3K/AKT pathway was deregulated in NPC cells overexpressed linc00312 in the presence of irradiation. This was confirmed by western blot with a reduction of p-AKT Ser473 in linc00312 overexpression + IR group. ATM is a major determinant of full activation of AKT through binding to AKT and mediating AKT Ser473 phosphorylation in response to ionizing radiation^34^. AKT regulates DNA-PKcs by facilitating the accumulation of DNA-PKcs at DNA damage sites, promoting DNA-PKcs kinase activity, and inducing autophosphorylation of DNA-PKcs at Ser2056, which is an essential step in DNA–PK disassembly from DSB sites and DNA-end ligation^35^. Therefore, we suggested that the inhibition effect of DNA-PKcs by linc00312 may be partially due to suppression of ATM and AKT activation.

**Fig. 6:**
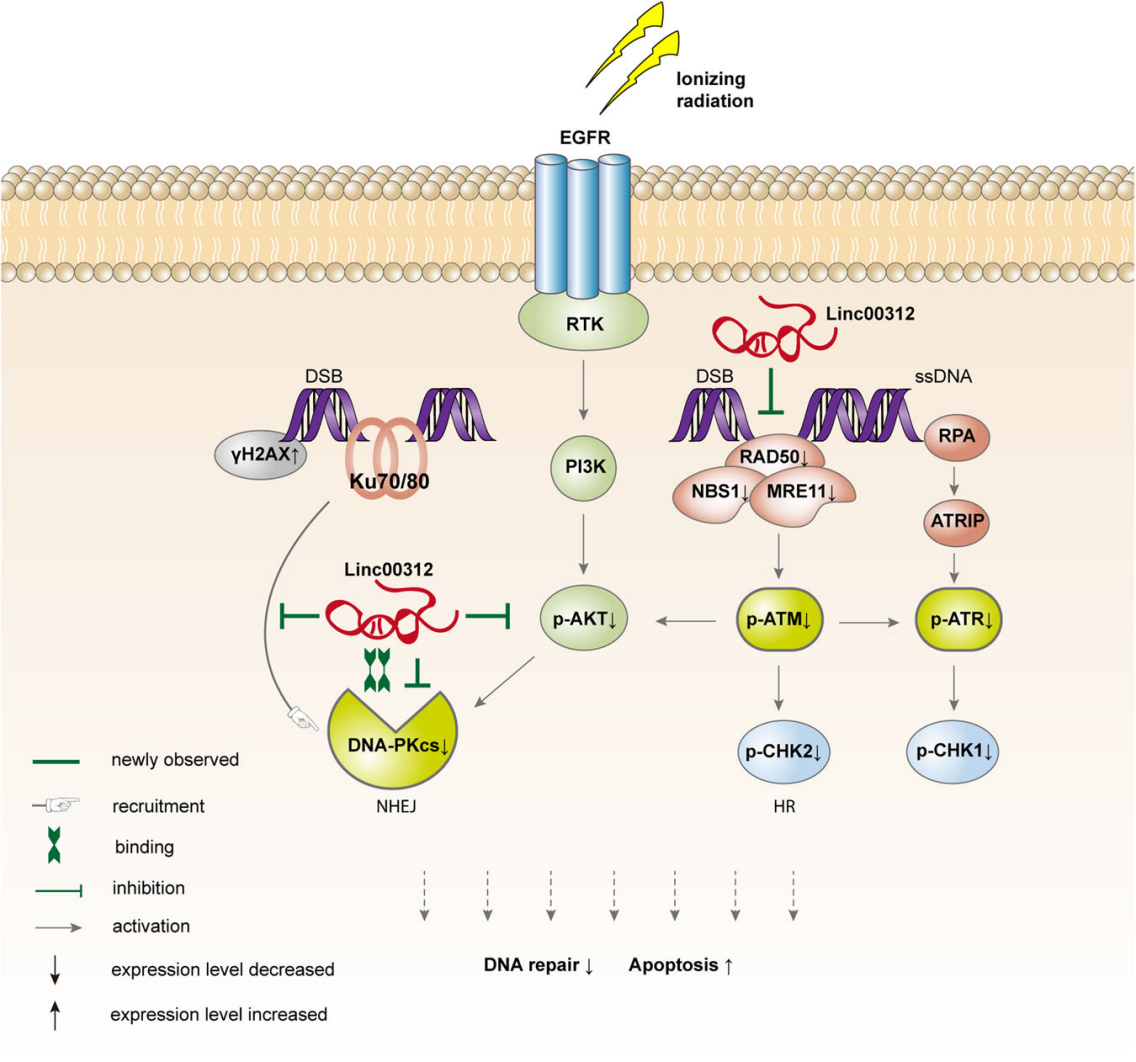
The role of linc00312 in regulating radiosensitivity of NPC. linc00312 directly binds to DNA-PKcs, hinders the recruitment of DNA-PKcs to Ku80, and inhibits phosphorylation of AKT–DNA–PKcs axis, therefore inhibiting the DNA damage signal sensation and transduction in the NHEJ repair pathway. In addition, linc00312 impairs DNA repair and cell cycle control by suppressing MRN–ATM–CHK2 signal and ATR–CHK1 signal.

Intracellular radiosensitivity is determined by the ability to repair DNA damage or to undergo apoptosis. The ionizing radiation-induced DNA lesion is recognized by sensor proteins (Ku70/Ku80, MRN, ATRIP) and amplified by signaling transducers (DNA-PKcs, ATM, ATR), which ultimately activates downstream effectors to modulate cell fate by handling DNA damage repair, cell cycle progression, and apoptosis. Given that ATM, ATR, and DNA-PKcs all belong to the PIKK family with similar kinase domain organizations and various common structural features, they can share certain substrates and have some overlapping functions^36^. A growing body of evidence has shown a cross-talk among ATM, ATR, and DNA-PKcs. As discussed earlier, ATM-dependent DNA-end resection provides the RPA-ssDNA signal for ATR recruitment and activation. ATM-mediated phosphorylation of DNA-PKcs is essential for DNA-PKcs function in DNA repair^37^. DNA-PKcs can also directly phosphorylate ATM at multiple sites and hence restrain its activity^38^. The role of linc00312 in NPC radiosensitivity regulation and DSB repair is summarized in Fig. 6.

## Conclusion

To the best of our knowledge, this is the first study that explains the radiosensitization effect of linc00312 in NPC. Our study demonstrates that linc00312 binds to DNA-PKcs and inhibits its recruitment to Ku-DNA. linc00312 inhibits irradiation-induced AKT– DNA–PKcs, MRN–ATM–CHK2, and ATR–CHK1 signal transduction, resulting in impaired DNA damage sensing, processing, and repairing ability, and leads to increased sensitivity to radiation treatments. Our study provides new insights into linc00312 in the regulating radiosensitivity in NPC.

## Materials and methods

### Cell lines and cell culture

The human immortalized normal nasopharyngeal epithelial cell line NP69 and nasopharyngeal carcinoma (NPC) cell lines CNE1, CNE2, HNE1, and HONE1 were obtained from the Advanced Research Center of Central South University (Changsha, Hunan, China). CNE1, CNE2, HNE1, and HONE1 cells were maintained in RPMI-1640 medium (Invitrogen, Carlsbad, CA, USA) supplemented with 10% fetal bovine serum (Invitrogen, Carlsbad, CA, USA) and 1% penicillin–streptomycin (Life Technologies, Carlsbad, CA, USA). NP69 cells were maintained in K-SFM medium (Invitrogen, Carlsbad, CA, USA) supplemented with 10% fetal bovine serum and 1% penicillin–streptomycin. All the cells were cultured in a humidified incubator with 5% CO_2_ at 37 °C. The cell lines were authenticated by DNA fingerprinting analysis using short-tandem repeat (STR) markers before the start of this study.

### Clinical specimens

Between 2015 and 2016, 92 newly diagnosed NPC tissue biopsies and 10 chronic inflammation of nasopharyngeal mucosa tissue biopsies were collected from patients receiving pharyngorhinoscopy inspection at The Affiliated Cancer Hospital of Xiangya School of Medicine. The specimens were immersed in RNAlater stabilization solution (Qiagen, Hilden, Germany) and were immediately frozen in liquid nitrogen. No patients received anticancer treatments before their operations. All patients received platinum-based induction chemotherapy plus concurrent radiochemotherapy after biopsy, and the therapeutic effects were evaluated by magnetic resonance imaging (MRI) 3 months after radiotherapy. Clinical efficacy was assessed according to Response Evaluation Criteria Solid Tumors (RECIST 1.1). We defined complete response (CR) and partial response (PR) as radiosensitive groups, while stable disease (SD) and progressed disease (PD) were defined as radioresistance groups. The total dose of radiotherapy was administered for 68–72 Gy (2 Gy/fraction, 5 days/week, followed by rest for 2 days). The neck received 60 Gy for lymph node-negative cases and 70 Gy for lymph node-positive cases. This study was approved by the Independent Ethical Committee of Institute of Clinical Pharmacology, Central South University (CTXY-140007-2), and written informed consent was obtained from all participants. The research was carried out according to the World Medical Association Declaration of Helsinki.

### RNA isolation and real-time PCR

Total RNA was extracted and purified from tissues/cells with RNAiso reagent (Takara, Japan). RNA was reverse transcribed to complementary DNA (cDNA) by using a PrimeScript^TM^ RT reagent kit (Takara, Japan). Real-time PCR was performed in Roche LightCycler 480 system (Roche, Basel, Switzerland) using a SYBR Premix Ex Taq^TM^ Kit (Takara, Japan). The 2^−ΔΔCt^ method was used to calculate the relative expression of the target gene. The primer sequences were listed as follows: GAPDH forward: 5′-ACAACTTTGGTATCGTGGAAGG-3′ and reverse: 5′-GCCATCACGCCACAGTTTC-3′; linc00312 forward: 5′-GATCTATGGCCCATCATTCTTT-3′ and reverse: 5′-GTCCATCATGTAGCAAGCAGT-3′.

### Plasmid and lentiviral vector construction

To construct the linc00312 overexpression vector, the full length of human linc00312 cDNA was directly synthesized by Genechem Company (Genechem, Shanghai, China) and cloned into pcDNA 3.1 plasmid. linc00312 overexpression lentiviral vector GV348 and control lentiviral vector were constructed and packed by Genechem Company.

### Isolation of cytoplasmic and nuclear RNA

Cytoplasmic and nuclear RNA were separated and purified using the PARIS Kit (Life Technologies, Carlsbad, CA, USA) following the manufacturer’s instructions. Briefly, cells were incubated with 0.5 mL cell fractionation buffer for 5 min on ice and then the homogenate was centrifuged for 5 min at 500*g* at 4 °C. The cytoplasmic fraction was in the supernatant and subsequently exposed to cell disruption buffer, while the nuclear fraction was in the pellet. The RNA levels of the nuclear and the cytoplasmic fractions were analyzed by RT-PCR.

### Fluorescence in situ hybridization

RNA in situ hybridization was performed using a Ribo^TM^ lncRNA FISH Probe Mix Kit (RiboBio, Guangzhou, China). The cells were seeded in a special laser confocal dish and fixed with 4% paraformaldehyde for 10 min, permeabilized with 0.5% Triton X-100 for 5 min, and washed with phosphate buffer saline (PBS) for three times at room temperature. The fixed cells were immersed in pre-hybridization buffer for 30 min at 37 °C and then incubated with target probes hybridization buffer overnight at 37 °C. The cells were washed by saline sodium citrate buffer and counterstained with 4′6-diamino-2-phenylindole (DAPI). Finally, the images were viewed by a laser scanning confocal microscope.

### Cell proliferation assay

The cells transfected with linc00312 overexpression vector or control vector were seeded into 96-well plates (1000 cells per well) and incubated for 24, 48, 72, and 96 h respectively. The Cell Counting Kit-8 (CCK-8) (Selleck, USA) was used to determine cell proliferation following the manufacturer’s protocol. Cell viability was assessed by the absorbance of each well at a wavelength of 450 nm by a spectrophotometer. Six replicate wells were prepared for each group, and three independent experiments were performed.

### Cell cycle progression

Cells were harvested and resuspended in pre-cooled 70% ethanol, and then stored overnight at −20 °C. After centrifugation and washing by PBS, the cells were resuspended in 0.5 mL propidium staining buffer containing RNase and incubated at 37 °C for 30 min. Cell cycle analysis was carried out using BD FACSDiva and FlowJo software.

### Hoechst staining

Cell apoptosis was measured by Hoechst staining when the cells reached a confluence of 80%. The cells were washed by PBS and fixed with 4% paraformaldehyde for 10 min. About 0.5 mL Hoechst 33258 staining solution was added into the cells for incubation for 5 min and then washed twice with PBS. Images were visualized using a fluorescence microscope.

### Annexin V-FITC cell apoptosis assay

The cells were transfected with linc00312 overexpression vector or control vector for 24 h and then exposed to 8 Gy irradiation. The cells were harvested at 0 h (before radiation) and 48 h after irradiation and then resuspended in the binding buffer and stained with FITC-Annexin V (AV) and propidium iodide (PI) according to the protocols (Beyotime, China). Cell apoptosis was immediately analyzed with a flow cytometry (BD Biosciences, San Jose, CA, USA) equipped with BD CellQuest software.

### Radiosensitivity assay

Radiosensitivity of HNE1 and HONE1 cells was assessed by clonogenic survival assay. Cells were plated in a 6-well plate and exposed to 0, 2, 4, 6, and 8 Gy ionizing radiation (IR), respectively, the next day. The surviving cells formed cell colonies (>50 cells/colony) 2 weeks after radiation, and were fixed with 4% paraformaldehyde and stained with 0.5% crystal violet. The cell dose–survival curve was fitted using the single-hit multitarget model: *y* = (1 − *e*^−D/D0^)^*n*^ in Graphpad Prism 6 software. The plating efficiency (PE) was defined as the number of colonies / the number of seeded cells × 100%. The survival fraction (SF) is the number of colonies at one irradiation dose divided by the number of colonies with a correction for the PE. The radiation protection factor (RPF) was calculated by dividing the AUC of the test cells by the AUC of control cells.

### Comet assay

Cells were transfected with linc00312 overexpression vector or control vector for 24 h and then exposed to 8 Gy irradiation. The cells were gently harvested at 0 h (before radiation) and 1, 4, 10, and 24 h after irradiation to yield approximately 1 × 10^6^ cells/mL; 10 μL cell suspension was added to 75 μL of 0.8% low-melting agarose at 37 °C, and layered onto the slide which was pre-coated with 1% normal melting agarose and kept at 4 °C for 10 min. Then, this slide was coated with 85 μL of 0.5% low-melting agarose at 37 °C. Slides were kept in the alkaline buffer for 20 min to allow unwinding of the DNA. Subsequently, slides were incubated in electrophoresis buffer and run at 300 mA, 25 V for 20 min using a comet assay tank. The slides were then neutralized with Tris-HCl buffer and dyed with 50 µL of 30 µg/mL EB for 20 min in the dark to stain DNA. Images were visualized using fluorescence microscope and quantification of tail moment and tail DNA was performed with Comet Assay Software.

### Immunofluorescence

The cells were seeded in a special laser confocal dish and transfected with linc00312 overexpression vector or control vector for 48 h. Then, 2 h after a total dose of 8 Gy irradiation, the cells were fixed with fixative solution for 15 min, permeabilized with permeabilization solution for 15 min, and blocked with blocking buffer for 60 min at room temperature using the Image-iT^TM^ Fixation/ Permeabilization Kit (Invitrogen, Carlsbad, CA, USA). The cells were incubated with a specific primary antibody (anti-DNA-PKcs, abcam, Cat: ab32566; anti-phospho-DNA-PKcs Ser2056, abcam, Cat: ab18192; anti-Ku80, abcam, Cat: ab119935) at 4 °C overnight and then labeled with Invitrogen Alexa Fluor Plus 594 goat anti-rabbit IgG secondary antibody or Alexa Fluor Plus 488 goat anti-mouse IgG secondary antibody for 1 h at room temperature. Nuclei were stained with Invitrogen ProLong™ glass antifade mountant with NucBlue™ Stain. Finally, cell images were captured by LSM 900 (Zeiss, Jena, Germany) confocal microscope using a 63× oil immersion objective lens with Airyscan imaging mode. The co-localization was analyzed by Zen blue software.

### Luciferase DNA repair reporter assays

pGL3 vector (Promega) was linearized using HindIII enzyme to generate the double strand break. HNE1 and HONE1 cells were transfected with linc00312 overexpression vector or control vector for 24 h using Lipofectamine 3000 (Life Technologies, Carlsbad, CA, USA). Then, the cells were transfected with 1 μg of either the linearized plasmid or uncut pGL3 control plasmid, along with 50 ng of Renilla luciferase vector as a transfection efficiency control. Luciferase activity was assayed using the Dual Luciferase Reporter Assay Kit (Promega) 24 h after transfection. In each sample, firefly luciferase activity was normalized to the Renilla luciferase signal. The percent reactivation of NHEJ was calculated as a ratio by normalizing linearized pGL3 to uncut pGL3 control. Data are presented as relative repair efficiencies, where percent reactivation from the experimental condition is normalized to the control condition.

### RNA pull-down assay

RNA pull-down assay was conducted with the BersinBio^TM^ RNA pulldown Kit (BersinBio, Guangzhou, China) following the manufacturer’s instructions. Briefly, 3 µg of biotin-labeled linc00312 or lacZ control probe was denatured at 90 °C for 2 min and mixed with RNA structure buffer for 20 min to format a RNA secondary structure. Next, the biotin-labeled probes were incubated with precleared human HNE1 whole-cell lysate in RIPA buffer and streptavidin magnetic beads at room temperature for 2 h. The tube was placed on a magnetic stand for 1 min to collect the beads, and the supernatant was removed and discarded. The beads were washed by protein elution buffer at room temperature for 2 h and the retrieved proteins were detected by mass spectrometry identification. Protein scores are derived from ion scores as a non-probabilistic basis for ranking protein hits. Ions score is −10*Log(*P*), where *P* is the probability that the observed match is a random event. Individual ion scores >23 indicate identity or extensive homology (*P* < 0.05).

### RNA immunoprecipitation (RIP)

RNA-binding protein immunoprecipitation (RIP) assay was performed using the BersinBio^TM^ RNA Immunoprecipitation (RIP) Kit (BersinBio, Guangzhou, China) according to the manufacturer’s instructions. Briefly, cells were lysed and incubated with protein A/G magnetic beads at 4 °C overnight and then mixed with anti-DNA-PKcs antibody (abcam, Cat: ab70250) or negative control IgG for 1 h at 4 °C. The beads were washed three times through a magnetic stand using polysome washing buffer. Next, the beads were resuspended in elution buffer and proteinase K and incubated at 55 °C for 1 h to extract the immunoprecipitated RNA. The purified RNA was determined by real-time RT-PCR and agarose gel electrophoresis.

### Microarray analysis

Cells were transfected with linc00312 overexpression vector or control vector for 24 h and then exposed to 8 Gy irradiation; 24 h after irradiation, the total RNA was extracted and hybridized to Affymetrix SurePrint G3 Human Gene Expression 8x60K v2 Microarray, which is designed for the global expression profiling of 6467 human lncRNA and 44,132 mRNA transcripts. The acquired array image data were preprocessed and analyzed using Feature Extraction software, and then the GeneSpring GX software (Agilent) was used for data summarization, normalization, and quality control. Cluster 3.0 software was applied for clustering analysis and graphical presentation. We set threshold values of ≥1.3 and ≤ −1.3 fold change and Benjamini-Hochberg-corrected *P* value of 0.05 to select the differentially expressed genes. GO enrichment analysis and KEGG pathway analysis were performed for the differential expression mRNAs.

### Western blotting

Cells were lysed in RIPA buffer containing 1% PMSF and phosphatase inhibitor cocktail buffer (Beyotime, China). Proteins were loaded onto SDS-PAGE gel for electrophoresis and then transferred onto a PVDF membrane. The blots were probed with primary antibodies at 4 °C overnight followed by incubation with secondary antibodies at room temperature for 1 h. The signal was visualized using enhanced chemiluminescence (Invitrogen, Carlsbad, CA, USA) and quantified by densitometry using Image Lab software. The primary antibodies were listed as follows: anti-GAPDH (Cell Signaling Technology (CST), Cat: 2118); anti-DNA-PKcs (abcam, Cat: ab70250); anti-ATM (CST, Cat: 2873); anti-ATM (Ser1981) (CST, Cat: 5883); anti-ATR (Proteintech, Cat: 19787-1-AP); anti-ATR (Ser428) (CST, Cat:2853); anti-Rad50 (CST, Cat: 3427); anti-NBS1 (Proteintech, Cat: 55025-1-AP); anti-Mre11 (CST, Cat: 4847); anti-Ku80 (Proteintech, Cat: 16389-1-AP); anti-Ku70 (Proteintech, Cat: 10723-1-AP); anti-Akt (CST, Cat: 9272); anti-Akt (Ser473) (CST, Cat: 9271); anti-Chk2 (Thr68) (CST, Cat: 2661); anti-Chk1 (Ser345) (CST, Cat: 2348); anti-PARP (CST, Cat: 9532); anti-Bcl-2 (CST, Cat:15071); anti-Bax (CST, Cat:5023); and anti-γH2AX (ABclonal, Cat: AP0099).

### Animal experiments

To determine the effects of linc00312 on NPC radiosensitivity in vivo, 1 × 10^7^ HNE1 cells stably expressing linc00312 or an empty vector were subcutaneously injected into the flank area of 5-week-old female immune deficient nude mice (BALB/c, SLAC Laboratory, Shanghai, China). About 3 weeks after cell incubation, the transplanted tumor xenografts reached approximately 50 mm^3^ and the mice were randomly divided into four treatment groups (*n* = 5/group): control group, linc00312 overexpression group, control + IR (6 Gy) group, and linc00312 overexpression + IR (6 Gy) group. Mice in the control + IR (6 Gy) group and linc00312 overexpression + IR (6 Gy) group were treated with a total dose of 6 Gy (3 Gy/day, 2 days in a row) X-radiation. Tumors were measured every 3 days and volume was calculated using the following formula: *V* = length × width × width/2. The mice were sacrificed when tumor volume reached 1000 mm^3^ or 24 days after treatment. Tumor specimens from nude mice were fixed in 4% paraformaldehyde and then embedded in paraffin. Sections were used for the immunohistochemical analysis of γH2AX and apoptosis analysis by TUNEL assay kits (Beyotime, China).

### Statistical analysis

All continuous variables were represented as means ± standard deviations, and categorical data were expressed as percentages. Student’s *t*-test was used to evaluate significant differences between the two groups for continuous variables. A two-tailed *P* value of < 0.05 was considered statistically significant. Data were analyzed by SPSS 16.0 software (SPSS, Chicago, IL, USA) and GraphPad Prism 5 (GraphPad, San Diego, CA, USA).

## Supplementary information

Supplementary Fig 1.

Supplementary Fig 1 legend.
