# Supplementary figures and images for: LncRNA linc00312 suppresses radiotherapy resistance by targeting DNA-PKcs and impairing DNA damage repair in nasopharyngeal carcinoma
Source: Cell Death Dis. 2021 Jan 4;12(1):69. doi: 10.1038/s41419-020-03302-2 (PMC7801696; doi:10.1038/s41419-020-03302-2)

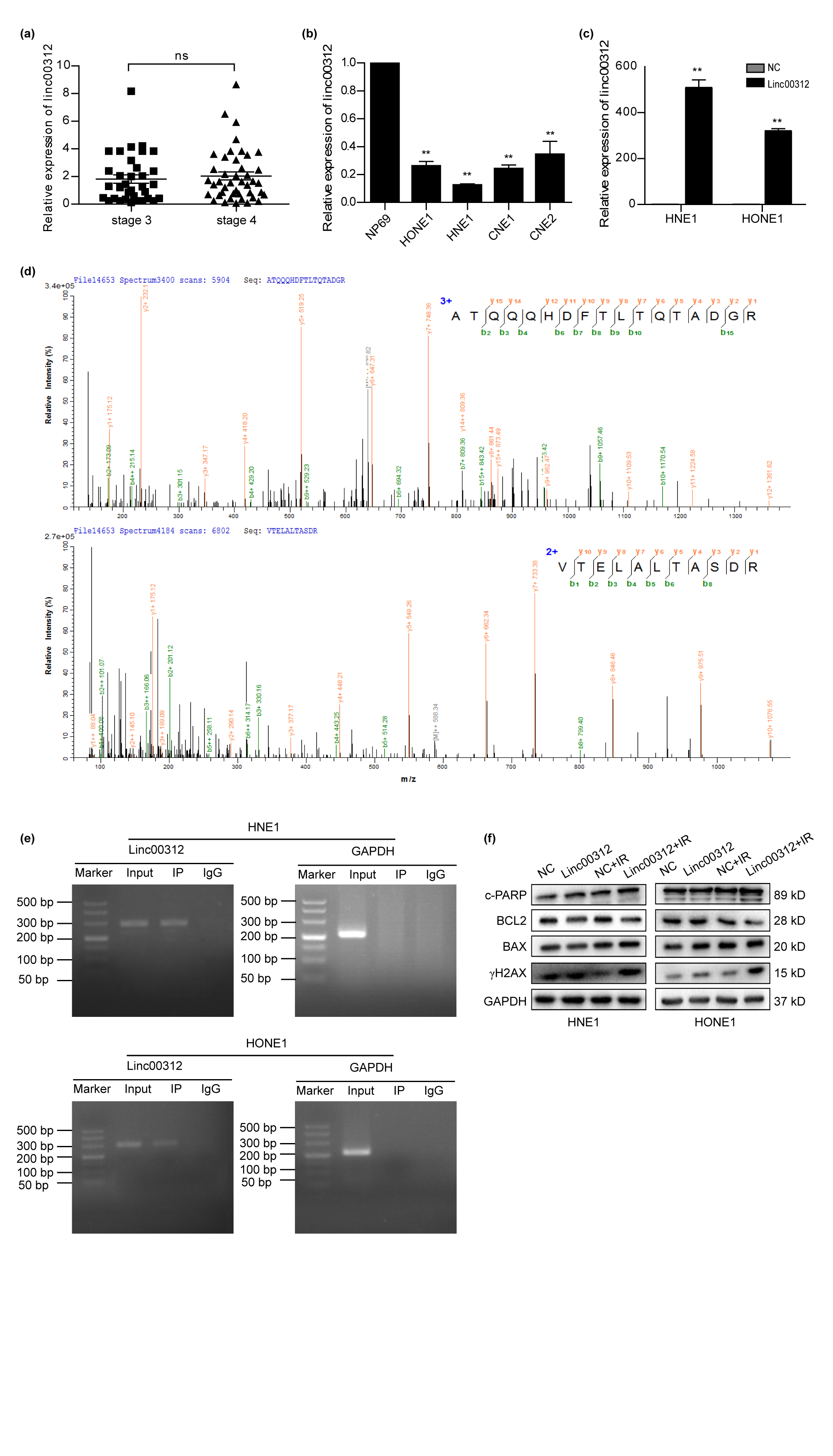

Supplement: Supplementary file 1 — Supplementary Fig 1. [file 41419_2020_3302_MOESM1_ESM.tif]
