# Supplementary material for: LncRNA linc00312 suppresses radiotherapy resistance by targeting DNA-PKcs and impairing DNA damage repair in nasopharyngeal carcinoma
Source: Cell Death Dis. 2021 Jan 4;12(1):69. doi: 10.1038/s41419-020-03302-2 (PMC7801696; doi:10.1038/s41419-020-03302-2)
Supplement: Supplementary file 2 — Supplementary Fig 1 legend. [file 41419_2020_3302_MOESM2_ESM.docx]

**Supplementary Fig 1 legend**

**(a)** Relative expression of linc00312 in NPC patients’ tissues with different clinical stages. **(b)** Relative expression of linc00312 in NPC cell lines (CNE1, CNE2, HONE1, HNE1) and normal nasopharyngeal epithelial cell line NP69. **(c)** The transfection efficiency of linc00312 overexpression vector in HNE1 and HONE1 cells was detected by RT-PCR. **(d)** The mass spectrogram of DNA-PKcs that pulled down by biotin-labeled linc00312 probe. **(e)** RIP assay was performed using DNA-PKcs specific antibody, and agarose gel electrophoresis was used to identify the IP products using linc00312 primers. **(f)** Western blotting of apoptosis-related proteins in linc00312 overexpressed cells or control cells with or without IR treatment. ^*^ *P* < 0.05, ^**^ *P* < 0.01.
